# Gefitinib in combination with prednisolone to avoid interstitial lung disease during non-small cell lung cancer treatment: A case report

**DOI:** 10.3892/ol.2013.1212

**Published:** 2013-02-27

**Authors:** XINYING XUE, QINGLIANG XUE, YUXIA LIU, LEI PAN, KAIFEI WANG, LINA ZHANG, NA WANG, BING YANG, JIANXIN WANG

**Affiliations:** 1 Cadres Respiratory Diseases Department of Beijing Shijitan Hospital, Beijing 100038;; 2 Research Department of Chinese Academy of Medical Sciences, Beijing 100864;; 3 Department of Respiratory Diseases of Chinese PLA General Hospital, Beijing 100853, P.R. China

**Keywords:** gefitinib, interstitial lung disease, non-small cell lung cancer, prednisolone, combination treatment

## Abstract

Gefitinib-induced interstitial lung disease (ILD) is a rare but lethal drug adverse event, which usually leads to the withdrawal of gefitinib and causes complications with anticancer treatment. In this study, gefitinib administration combined with prednisolone in a female with stage IIIb non-small cell lung cancer (NSCLC) produced a good outcome without inducing ILD. The results suggested that combined administration of gefitinib with glucocorticoids may be an efficient method to treat NSCLC while avoiding complications with ILD.

## Introduction

Gefitinib, an epidermal growth factor receptor (EGFR) tyrosine kinase inhibitor, is currently extensively used for the treatment of advanced non-small cell lung cancer (NSCLC). The drug exhibits efficacious anticancer activity, particularly in patients with clinical features including EGFR gene mutations, a history of never smoking, adenocarcinoma histology, female gender and Asian origin (1,2). The most common adverse effects of gefitinib are a skin rash and diarrhea, which are generally tolerable. However, interstitial lung disease (ILD) is occasionally induced by gefitinib, and is a serious and lethal adverse event (3,4) that usually leads to the withdrawal of gefitinib and causes comlications with anticancer treatment. Herein, we recommend implementing our successful gefitinib administration, which may be a potential method of solving this issue.

## Case report

**Figure 1:**
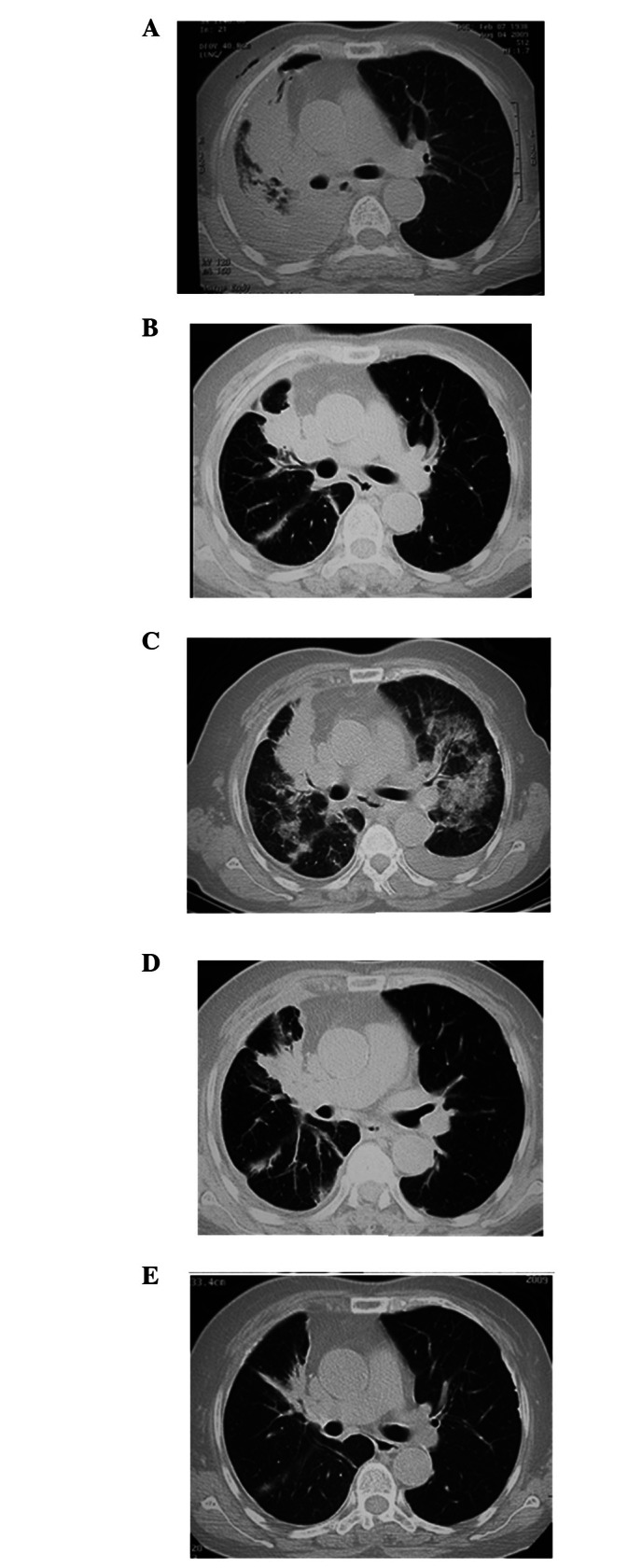
Chest computed tomography (CT) findings of the patient in this study. (A) Chest CT findings prior to gefitinib treatment reveal a mass in the right middle lobe with ipsilateral pleural effusion and atelectasis. (B) Chest CT findings on day 21 following gefitinib treatment reveal shrinkage of the mass in the right middle lobe, improved atelectasis and complete disappearance of pleural effusion. (C) On day 38 following gefitinib treatment, chest CT findings reveal new bilateral diffuse ground-glass shadows in addition to the findings in B. (D) The condition of the patient rapidly improved when methylprednisolone (240 mg/day) was administered for three days. (E) Chest CT findings following 18 months of gefitinib treatment revealed a significant shrinkage of the mass in the right middle lobe, and disappearance of atelectasis and pleural effusion.

The present case was a 71-year-old female diagnosed with stage IIIb non-small cell lung cancer (NSCLC). The study was approved by the Ethics Committee of the PLA General Hospital, Beijing, China. Written informed consent was obtained from the patient’s family. Chest computed tomography (CT) findings (Fig. 1A) revealed a mass in the right middle lobe with ipsilateral pleural effusion and atelectasis; therefore, chemotherapy was selected as the first-line treatment. However, two chemotherapy regimens with carboplatin and paclitaxel were inefficacious. Due to the fact that the patient possessed clinical features including a history of never smoking, adenocarcinoma histology, female gender and Asian ethnicity, gefitinib was chosen as the second-line treatment, even though the patient did not exhibit EGFR gene mutations. Following the administration of gefitinib (250 mg/day), the symptoms of the patient were gradually relieved. Further chest CT examinations were performed (Fig. 1B), revealing a shrinkage of the mass in the right middle lobe, improved atelectasis and complete disappearance of pleural effusion.

However, gefitinib also gave rise to certain adverse effects in the patient. From the 38th day following the onset of gefitinib, the patient began to complain of shortness of breath and a middle-grade fever. A chest auscultation revealed inspiratory crackles, while arterial blood gas analysis showed hypoxemia (pO_2_, 57.2 mmHg; pCO_2_, 35.1 mmHg), and sputum and blood cultures for microorganisms were negative. Chest CT was performed once more (Fig. 1C), revealing novel bilateral diffuse ground glass shadows in addition to the previous chest CT findings. Based on the aforementioned observations, the patient was suspected of having gefitinib-induced ILD. Thus, gefitinib was withdrawn and high-dose methylprednisolone (240 mg/day) was administered for three days. The condition of the patient rapidly improved (Fig. 1D) which supported the previous diagnosis of gefitinib-induced ILD. After three days, methylprednisolone was tapered and finally substituted by prednisolone. Due to the curative effect, and at the request of the patient and the patient’s family, gefitinib was administered again. However, this time, gefitinib was administered in combination with prednisolone (10 mg/day), and the dose of gefitinib was gradually increased from 250 mg every two days to 250 mg/day. During the 18 months of follow-up, the adverse event of ILD did not recur. Recent chest CT findings (Fig. 1E) revealed a significant shrinkage of the mass in the right middle lobe, and a disappearance of atelectasis and pleural effusion.

## Discussion

The pathogenesis of gefitinib-induced ILD is not fully understood. Possible mechanisms include increased apoptosis, inhibition of regeneration of pulmonary epithelium and endothelium, fibroblast proliferation and allergic reaction. The incidence of gefitinib-induced ILD is ∼1% worldwide and ∼4% in East Asia (5,6). Approximately one third of patients with gefitinib-induced ILD died. The median time to onset of gefitinib-associated ILD was 24 days in Japan and 42 days in America (7). To prevent exacerbation of ILD, early diagnosis and the withdrawal of gefitinib are required, and early administration of high-dose glucocorticoids generally presents curative effects. However, when gefitinib is administered again, ILD usually recurs (8), causing disruption to the anti-cancer treatment of patients with advanced NSCLC that do not benefit from chemotherapy. In this study, the administration of gefitinib combined with prednisolone not only prevented the recurrence of ILD, but also retained the anticancer activity of gefitinib, thus providing a method to solve the issue.

The risk factors of gefitinib-induced ILD, which are also associated with a poor prognosis, include a history of smoking, pre-existing interstitial pneumonia, reduced normal lung, old age and complications of cardiovascular diseases (9). Therefore, to prevent ILD when gefitinib is administered in patients with these risk factors, the use of a combination treatment with prednisolone should be considered.
